# Diagnostic utility of exome sequencing followed by research reanalysis in human brain malformations

**DOI:** 10.1093/braincomms/fcae056

**Published:** 2024-02-28

**Authors:** Daniz Kooshavar, David J Amor, Kirsten Boggs, Naomi Baker, Christopher Barnett, Michelle G de Silva, Samantha Edwards, Michael C Fahey, Justine E Marum, Penny Snell, Kiymet Bozaoglu, Kate Pope, Shekeeb S Mohammad, Kate Riney, Rani Sachdev, Ingrid E Scheffer, Sarah Schenscher, John Silberstein, Nicholas Smith, Melanie Tom, Tyson L Ware, Paul J Lockhart, Richard J Leventer

**Affiliations:** Murdoch Children’s Research Institute, Parkville, VIC 3052, Australia; Department of Paediatrics, The University of Melbourne, Parkville, VIC 3052, Australia; Murdoch Children’s Research Institute, Parkville, VIC 3052, Australia; Department of Paediatrics, The University of Melbourne, Parkville, VIC 3052, Australia; Centre for Clinical Genetics, Sydney Children’s Hospital, Randwick, NSW 2031, Australia; Department of Clinical Genetics, The Children’s Hospital Westmead, Westmead, NSW 2145, Australia; Australian Genomics, Parkville, VIC 3052, Australia; Victorian Clinical Genetics Services, Murdoch Children's Research Institute, Parkville, VIC 3052, Australia; SA Clinical Genetics Service, Women's and Children's Hospital, North Adelaide, SA 5006, Australia; Murdoch Children’s Research Institute, Parkville, VIC 3052, Australia; Australian Genomics, Parkville, VIC 3052, Australia; Harry Perkins Institute of Medical Research, University of Western Australia, Nedlands, WA 6009, Australia; Department of Paediatrics, Monash University, Clayton, VIC 3168, Australia; Australian Genomics, Parkville, VIC 3052, Australia; Murdoch Children’s Research Institute, Parkville, VIC 3052, Australia; Murdoch Children’s Research Institute, Parkville, VIC 3052, Australia; Department of Paediatrics, The University of Melbourne, Parkville, VIC 3052, Australia; Murdoch Children’s Research Institute, Parkville, VIC 3052, Australia; Department of Neurology, Westmead Hospital, Westmead, NSW 2145, Australia; Neurosciences Unit, Queensland Children’s Hospital, South Brisbane, QLD 4101, Australia; Faculty of Medicine, University of Queensland, St Lucia, QLD 4072, Australia; Centre for Clinical Genetics, Sydney Children’s Hospital, Randwick, NSW 2031, Australia; Murdoch Children’s Research Institute, Parkville, VIC 3052, Australia; Department of Paediatrics, The University of Melbourne, Parkville, VIC 3052, Australia; Department of Medicine, Epilepsy Research Centre, University of Melbourne, Austin Health and Florey Institute, Heidelberg, VIC 3084, Australia; Department of Neurology, The Royal Children's Hospital, Parkville, VIC 3052, Australia; Paediatric and Reproductive Genetics Unit, Women’s and Children’s Hospital, Adelaide, SA 5006 Australia; Department of Neurology, Princess Margaret Hospital, Nedlands, WA 6009, Australia; Department of Neurology and Clinical Neurophysiology, Women’s and Children’s Hospital, North Adelaide, SA 5006, Australia; Genetic Health Queensland, Royal Brisbane and Women’s Hospital, Herston, QLD 4029 Australia; Department of Paediatrics, Royal Hobart Hospital, Hobart, TAS 7000, Australia; Murdoch Children’s Research Institute, Parkville, VIC 3052, Australia; Department of Paediatrics, The University of Melbourne, Parkville, VIC 3052, Australia; Murdoch Children’s Research Institute, Parkville, VIC 3052, Australia; Department of Paediatrics, The University of Melbourne, Parkville, VIC 3052, Australia; Department of Neurology, The Royal Children's Hospital, Parkville, VIC 3052, Australia

**Keywords:** exome sequencing, brain malformations, genomics

## Abstract

This study aimed to determine the diagnostic yield of singleton exome sequencing and subsequent research-based trio exome analysis in children with a spectrum of brain malformations seen commonly in clinical practice. We recruited children ≤ 18 years old with a brain malformation diagnosed by magnetic resonance imaging and consistent with an established list of known genetic causes. Patients were ascertained nationally from eight tertiary paediatric centres as part of the Australian Genomics Brain Malformation Flagship. Chromosome microarray was required for all children, and those with pathogenic copy number changes were excluded. Cytomegalovirus polymerase chain reaction on neonatal blood spots was performed on all children with polymicrogyria with positive patients excluded. Singleton exome sequencing was performed through a diagnostic laboratory and analysed using a clinical exome sequencing pipeline. Undiagnosed patients were followed up in a research setting, including reanalysis of the singleton exome data and subsequent trio exome sequencing. A total of 102 children were recruited. Ten malformation subtypes were identified with the commonest being polymicrogyria (36%), pontocerebellar hypoplasia (14%), periventricular nodular heterotopia (11%), tubulinopathy (10%), lissencephaly (10%) and cortical dysplasia (9%). The overall diagnostic yield for the clinical singleton exome sequencing was 36%, which increased to 43% after research follow-up. The main source of increased diagnostic yield was the reanalysis of the singleton exome data to include newly discovered gene–disease associations. One additional diagnosis was made by trio exome sequencing. The highest phenotype-based diagnostic yields were for cobblestone malformation, tubulinopathy and lissencephaly and the lowest for cortical dysplasia and polymicrogyria. Pathogenic variants were identified in 32 genes, with variants in 6/32 genes occurring in more than one patient. The most frequent genetic diagnosis was pathogenic variants in *TUBA1A*. This study shows that over 40% of patients with common brain malformations have a genetic aetiology identified by exome sequencing. Periodic reanalysis of exome data to include newly identified genes was of greater value in increasing diagnostic yield than the expansion to trio exome. This study highlights the genetic and phenotypic heterogeneity of brain malformations, the importance of a multidisciplinary approach to diagnosis and the large number of patients that remain without a genetic diagnosis despite clinical exome sequencing and research reanalysis.

## Teaser

Kooshavar *et al*. report the yield of exome sequencing followed by research reanalysis in 102 children with brain malformations ascertained as part of the Australian Genomics Health Alliance. Ten phenotypes were ascertained with yield from singleton exome being 36% increasing to 43% with research reanalysis including expansion to trio exome.

## Graphical Abstract

**Graphical Abstract:**
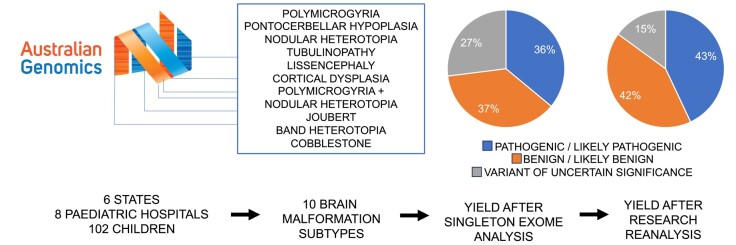

## Introduction

Human brain malformations comprise a broad spectrum of congenital anomalies that can be isolated or associated with malformations elsewhere in the body. Whilst some brain malformations are due to non-genetic causes, such as *in utero* infection or perfusion failure to the developing foetus, many are due to either known or presumed genetic disorders. The pathogenic variants are found in developmental genes, such as those required for neural tube formation, prosencephalic development, neuronal differentiation, neuronal migration or cortical organization.^1,2^ The advent and adoption of genomic sequencing have led to a rapid increase in the discovery of genetic causes of brain malformations and the ability to make a precise genetic diagnosis necessary for accurate genetic and prognostic counselling, including informing reproductive options.^3^ Whilst there are studies of the yield of genomic research testing in selected malformations such as lissencephaly, the diagnostic utility of clinical genomic testing applied to a broad range of brain malformations ascertained through routine clinical practice has not been determined.

Australian Genomics (formerly known as the Australian Genomics Health Alliance) is a federally funded, Australia-wide initiative that commenced in 2016 with the initial aim to provide evidence-based genomic medicine to improve health care outcomes cost-effectively.^4,5^ One of the initial focus areas of Australian Genomics was rare neurodevelopmental disorders, and one disease flagship focussed specifically on brain malformations. Here, we present the results from the Australian Genomics Brain Malformation Flagship, reporting the utility of clinical singleton exome sequencing (ES) followed by a research investigation of a cohort of 102 children with a range of well-phenotyped brain malformations. We describe the phenotypic spectrum, the diagnostic yield, and research follow-up, including reanalysis of the singleton ES data and trio ES for those patients who remained undiagnosed after singleton ES.

## Materials and methods

### Ascertainment

A network of paediatric neurologists and geneticists supported by genetic counsellors and project managers was established to cover all states and territories of Australia. The state flagship leads made discipline-specific recruitment calls to their colleagues and reviewed the clinical and imaging data of potential participants. Proforma and imaging data provided by the state flagship leads were reviewed by the national flagship lead (R.J.L.) and eligible participants were approved for recruitment to the study (see below). The state genetic counsellor obtained written informed consent from the parents or legal guardians of each patient for study participation and a blood-derived DNA sample from the proband and both parents where possible. Ethical approval was provided by the Human Research Ethics Committee at Melbourne Health (HREC/16/MH/251) consistent with the Declaration of Helsinki.

### Inclusion and exclusion criteria

Inclusion was restricted to a list of relatively homogeneous brain malformation phenotypes with a known list of monogenic causes. We chose this approach as the aim of the study was to determine diagnostic rates according to ‘real-world’ clinical practice in which exome data are analysed using suggested gene lists for the phenotype in question. The aim of this study was not gene discovery. The phenotypes included were cobblestone malformation, focal cortical dysplasia, holoprosencephaly, Joubert syndrome, lissencephaly, grey matter heterotopia, polymicrogyria/schizencephaly, pontocerebellar hypoplasia and subcortical band heterotopia. Further inclusion requirements comprised diagnosis by postnatal brain MRI showing a brain malformation of presumed genetic basis given the clinical and MRI features or family history. Before recruitment, all participants returned a non-diagnostic chromosome microarray. Patients with polymicrogyria/schizencephaly were required to have a negative cytomegalovirus PCR from the neonatal Guthrie card blood spot to be included. As the referring clinicians were all based in paediatric centres, recruitment age was restricted to 18 years or less. Patients were excluded where a non-genetic cause for the brain malformation was known or suspected based on the antenatal history (e.g. infection) or imaging appearance, a diagnosis already made by non-genetic means such as biochemical studies (e.g. abnormal very long chain fatty acids in suspected peroxisomal disorder) or where genomic testing (multi-gene panel, exome or genome) had already been performed.

### Genetic testing and analysis

#### Clinical singleton ES

Clinical singleton ES was performed on the probands’ peripheral blood-derived gDNA by a clinically accredited laboratory, Victorian Clinical Genetics Services, Melbourne, Australia. Coding regions of the genome were enriched using the SureSelect QXT Clinical Research Exome V1 or V2 (Agilent Technologies, Inc., CA, USA) capture kits and sequenced at targeted mean coverage of 100× using an Illumina (Illumina, Inc., CA, USA) instrument. A minimum of 90% of bases was sequenced to at least 15× coverage. Data were aligned to the reference genome (GRCh38), and the variants were called within the coding exons ± 8 bp using Cpipe.^6^ Variants were reported against the Human Genome Organization Gene Nomenclature Committee recommended transcript and according to Human Genome Variation Society nomenclature. The curation of variants was phenotype driven and performed according to the clinical genetics laboratory protocols. Variant prioritization was first performed on the gene lists (Supplementary Table 1) recommended by the clinician based on phenotypic presentation. Where no causative variants were identified within the prioritized genes, analysis was expanded to truncating and very rare/conserved missense variants identified in the Mendeliome.^7^ The candidate variants were reviewed at a multidisciplinary team (MDT) meeting comprised of clinicians, genomic laboratory staff and bioinformaticians and classified based on ACMG guidelines.^8^ On the advice of the MDT, for highly relevant variants where the gene matched the phenotype, the inheritance pattern was determined by segregation analysis if possible. Finally, the treating clinician and/or a genetic counsellor explained the clinical genetic findings to families.

#### Research reanalysis of the singleton ES

We reanalysed the ES data in a research setting for patients who had a negative result on clinical singleton ES. We used a research instance of seqr,^9^ implemented by Murdoch Children’s Research Institute, Melbourne, Australia, to search for single nucleotide variants and small deletions and insertions. The analysis included non-synonymous single nucleotide variants, small insertions and deletions and all modes of inheritance in the coding and the flanking regions with a read depth of at least 15×. We took a tier-based approach to analyse the variants by creating two gene lists. Tier 1 (Supplementary Table 2) consisted of 687 genes with strong evidence of association with brain malformations based on clinical gene lists,^10^ National Institutes of Health genetic testing registry^11^ and literature review. Tier 2 (Supplementary Table 3) comprised genes with some evidence of association with brain malformations, including genes related to genes in Tier 1 in terms of physical molecular interaction, expression, co-localization and shared protein domains and pathways. This tier was compiled using the Open Targets Platform online tool,^12^ an internally designed gene panel for neurological diseases, and the GeneMANIA server.^13^ If no strong candidates were detected in the two-tier analyses, the reanalysis was expanded to an exome-wide variant search. We also utilized CXGo^14^ to test for copy number variants (CNVs) within the ES data.

#### Variant filtering and prioritization

Variants were excluded when they met at least one of the below criteria: allele frequency in population databases (ExAC, gnomAD v2.1.1, gnomAD v3.1.2, TOPMed)^15^ greater than 0.001 for recessive conditions and 0.0005 for dominant/de-novo conditions and if there were reports of homozygous or hemizygous patients in population databases for recessive and X-linked diseases respectively; allelic balance below 0.15 for heterozygote variants; variants in low complexity regions of the genome, i.e. single base repeat regions and short tandem repeats; and variants with strand bias. However, only the allele frequency filter was applied as an initial filtering step, and the remaining filters were only used to rule out the variants in the analysis process on a case-by-case manner.

An experienced researcher performed the variant interpretation, and candidate variants were reviewed at an MDT meeting. The following databases and tools were used for variant prioritization: CADD,^16^ REVEL,^17^ PrimateAI,^18^ SpliceAI,^19^ Eigen,^20^ Polyphen,^21^ Sift,^22^ Mutation Taster,^23^ Fathmm,^24^ Metasvm,^25^ Gerp Rs,^26^ Phastcons 100 Vert,^27^ OMIM^®^, Clinvar, GTEx, Uniprot,^28^ decipher^29^ and literature search. Ultimately, the MDT decided if a candidate variant was consistent with the patient’s phenotype and causative for the disease.

### Parental ES and trio analysis in the research setting

Parental samples were obtained for available probands where reanalysis of the singleton ES data had not identified a pathogenic variant. ES was performed on parental DNA isolated from either peripheral blood or saliva swab. The data were analysed together with the proband’s ES data as a trio using seqr. The variant search was executed on Tier 1 and 2 gene lists and exome-wide following the method mentioned above. Variants were excluded if they were inherited from unaffected parents when considering autosomal dominant inheritance.

## Results

### Ascertainment and participant demographics

One hundred and sixty-four patients were referred for recruitment. After review, 62 were rejected as they did not meet the inclusion criteria. The most common reasons for rejection were imaging phenotype not consistent with inclusion criteria (30 patients), parent declining to participate (18 patients) and prior genetic/genomic testing (7 patients). The remaining102 patients proceeded to ES. Patients were recruited from all Australian states and territories, with the majority from the three most populous states of New South Wales (28), Victoria (36) and Queensland (21), respectively. There were 48 females and 54 males. The mean age at ES was 5.4 years.

### Imaging diagnosis

Ten imaging phenotypes were ascertained. These were polymicrogyria (37), pontocerebellar hypoplasia (14), grey matter heterotopia (11), tubulinopathy (10), lissencephaly (10), focal cortical dysplasia (9), polymicrogyria + grey matter heterotopia (5), Joubert syndrome (3), subcortical band heterotopia (2) and cobblestone malformation (1). The imaging phenotype of ‘tubulinopathy’ was recognized soon after the study commenced with patients with this phenotype having been initially referred with a diagnosis of either polymicrogyria or lissencephaly. No patients with holoprosencephaly were ascertained. None of the patients with focal cortical dysplasia had hemispheric lesions such as hemimegalencephaly or hemispheric dysplasia or megalencephaly syndromes. Within each of these imaging phenotypes, there was a spectrum of findings, with some patients having additional brain malformations such as callosal dysgenesis or intracranial cysts.

### Clinical singleton ES diagnostic yield

Clinical singleton ES yielded a diagnosis in 36% (37/102) of patients (Table 1). Among patients with a genetic diagnosis, 8/37 (22%) had pathogenic variants in *TUBA1A*. *DCX*, *TUBB2B*, *DYNC1H1*, *FLNA* and *FOXG1* were identified as the causative gene in two patients each. The remaining 19 genetic diagnoses were made in single cases. 59% (22/37) of the genetic diagnoses followed autosomal dominant inheritance. Nine patients had autosomal recessive disorders with four being compound heterozygous, and five homozygous. Inheritance was consistent with X-linked dominant in five patients and X-linked recessive in one patient. 51% (22/41) of the identified variants were novel.

**Table 1:** Results of the clinical singleton ES analysis

ID	Sex	MRI^a^	Gene	Zyg	Seg	Variant(s)	Cl	Ref
A1422012	M	TUB	TUBA1A	Het	De novo	NM_006009.3:c.3+3delG	LP	-
A0122003	M	TUB	TUBA1A	Het	ND	NM_006009.3:c.236G>C, p.(Arg79Pro)	LP	-
A0122026	F	TUB	TUBA1A	Het	ND	NM_006009.4:c.652G>A, p.(Asp218Asn)	P	^30^
A1422016	F	PMG	TUBA1A	Het	De novo	NM_006009.3:c.790C>T, p.(Arg264Cys)	P	^31^
A1422006	M	TUB	TUBA1A	Het	De novo	NM_006009.3:c.992C>A, p.(Ala331Asp)	LP	-
A1122010	M	TUB	TUBA1A	Het	de novo	NM_006009.3:c.1007A>G, p.(Lys336Arg)	P	-
A1122001	F	LIS	TUBA1A	Het	De novo	NM_006009.3:c.1204C>T, p.(Arg402Cys)	P	^32^
A0322003	M	TUB	TUBA1A	Het	De novo	NM_006009.4:c.1265G>A, p.(Arg422His)	P	^33^
A0322002	F	SBH	DCX	Het	De novo	NM_178151.2:c.272T>C, p.(Leu91Pro)	LP	^34^
A0122009	M	LIS	DCX	Hemi	Maternal	NM_178151.2:c.587G>A, p.(Arg196His)	P	^35^
A1422004	M	TUB	TUBB2B	Het	De novo	NM_178012.4:c.523G>T, p.(Val175Leu)	LP	-
A1422013	M	TUB	TUBB2B	Het	De novo	NM_178012.4:c.989T>C, p.(Met330Thr)	LP	-
A1422007	M	LIS	DYNC1H1	Het	De novo	NM_001376.4:c.926G>A, p.(Arg309His)	P	^36^
A0422007	M	LIS, MLIS	DYNC1H1	Het	ND	NM_001376.5:c.3603G>T, p.(Arg1201Ser)	LP	^37^
A0122025	F	PNH	FLNA	Het	ND	NM_001456.3:c.3571_3577del, p.(Ala1191*)	P	-
A0122016	F	PNH	FLNA	Het	De novo	NM_001456.3:c.4866C>G, p.(Tyr1622*)	P	-
A0122027	M	PMG	FOXG1	Het	ND	NM_005249.4:c.561C>G, p.(Asn187Lys)	P	^38^
A0722005	F	PMG	FOXG1	Het	ND	NM_005249.4:c.1007dup, p.(Ser366Argfs*119)	P	-
A0122001	F	JBR	TMEM237	Het	Com het	NM_001044385.2:c.1066dupC, p.(Gln356Profs*24)	P	^39^
			TMEM237	Het		NM_001044385.2:c.1038-1G>C	LP	-
A0122005	M	PNH PMG	NSDHL	Hemi	ND	NM_001129765.1:c.982C>T, p.(Arg328Trp)	LP	-
A0122007	M	PCH	CHMP1A	Hom	ND	NM_002768.4:c.34delG, p.(Ala12Argfs*22)	LP	-
A0122011	F	PCH	COL4A1	Het	ND	NM_001845.4:c.1990+1G>A	LP	^40^
A0122013	F	PCH	PIGG	Het	Com het	NM_001127178.2:c.2624_2625del, p.(Leu875*)	P	-
			PIGG	Het		NM_001127178.2:c.2326G>T, p.(Asp776Tyr)	LP	-
A0122014	F	PMG	ACTB	Het	De novo	NM_001101.4:c.193C>T, p.(Leu65Phe)	LP	^41^
A0122018	F	PCH	CASK	Het	De novo	NM_003688.3:c.1969_1971del, p.(Trp657del)	LP	-
A0122029	M	PMG	SON	Het	ND	NM_138927.2:c.3852_3856del, p.(Met1284Ilefs*2)	P	^42^
A0422004	M	PCH	KIF1A	Het	De novo	NM_004321.7:c760C>T, p.(Arg254Trp)	P	^43^
A0822006	F	JBR	AHI1	Hom	ND	NM_017651.4:c.2495del, p.(Leu832*)	P	^44^
A0822007	F	LIS, PACC VH	ZBTB18	Het	De novo	NM_205768.2:c.1347C>G, p.(Cys449Trp)	LP	-
A1022001	M	PCH	TSEN54	Het	ND	NM_207346.2:c.973del, p.(Ala325Profs*17)	P	-
			TSEN54	Het		NM_207346.2:c.919G>T, p.(Ala307Ser)	P	^45^
A1122004	F	FCD	DEPDC5	Het	ND	NM_001242896.2:c.1264C>T, p.(Arg422*)	P	^46^
A1122013	F	COB	POMGNT1	Het	Com het	NM_017739.3:c.1342G>C, p.(Gly448Arg)	P	^47^
			POMGNT1	Het		NM_017739.3:c.636C>T, p.(Asp179Valfs*23)	P	^48^
A1422005	F	PMG	KIF1BP	Hom	Maternal, Paternal	NM_015634.3:c.1520-1523delATAA, p.(Asn507Ilefs*7)	LP	-
A1422009	M	TUB	TUBB2A	Het	De novo	NM_001069.2:c.1070C>T, p.(Pro357Leu)	LP	-
A1422010	F	PCH	NIPBL	Het	De novo	NM_133433.3:c.1084_1088dupCTTTC, p.(Arg364Phefs*4)	P	-
A0722004	F	PNH	DCHS1	Hom	ND	NM_003737.3:c.7204G>A, p.(Asp2402Asn)	LP	-
A0122031	F	LIS	WDR62	Hom	ND	NM_001083961.1:c.4397T>A, p.(Leu1466Gln)	LP	-

Human reference genome used is GRCh38.

Cl, pathogenicity classification; COB, cobblestone malformation; Com het, compound heterozygote; F, female; FCD, focal cortical dysplasia; Hemi, hemizygote; Het, heterozygote; Hom, Homozygote; JBR, Joubert syndrome; LIS, lissencephaly; LP, likely pathogenic; M, male; MLIS, microlissencephaly; ND, not determined; P, pathogenic; PACC, partial agenesis of corpus callosum; PCH, pontocerebellar hypoplasia; PMG, polymicrogyria; PNH, periventricular nodular heterotopia; Ref, reference; SBH, subcortical band heterotopia; Seg, segregation; TUB, tubulinopathies; VH, vermis hypoplasia; WM, white matter abnormality; Zyg, zygosity.

^a^MRI diagnosis: Anything after the comma is the additional MRI diagnosis.

### Research diagnostic yield

Parents for 65% (42/65) of genetically undiagnosed patients consented to research trio analysis. Research reanalysis of the singleton ES together with trio analysis in cases that remained undiagnosed after clinical singleton ES, resulted in causative variants being identified in seven additional patients in *ACO2*, *AP4B1, ATP1A3*, *CEP85L* and *RELN* (Table 2). Two CNVs were identified by CXGO, including a one-exon deletion in *PAFAH1B1* and a deletion encompassing parts of *KDM4A*, *KDM4A-AS1* and *ST3GAL3*. The multi-gene deletion and the *ACO2* variant were curated as variants of uncertain significance; however, based on phenotype match and clinical presentation, the MDT concluded that they either contributed to or caused the disease in these patients. The final diagnostic yield was increased to 43% (44/102) following the research analysis.

**Table 2:** Results of the research ES analysis

ID	Sex	MRI	Gene	Zyg	Seg	Variant(s)	Cl	Ref
A0422001	M	PMG	AP4B1	Het	Com het	NM_001253852.3:c.1540C>T, p.(Arg514Ter)	P	^49^
			AP4B1	Het		NM_001253852.3:c.1216C>T, p.(Arg406Ter)	P	^50^
A0422002	M	PMG	ATP1A3	Het	De novo	NM_152296.5:c.2570_2572del, p.(Phe857del)	LP	^51^
A0722001	F	LIS	PAFAH1B1	Het	De novo	NM_000430.4:deletion of exon 10	P	-
A1122003	M	LIS	CEP85L	Het	De novo	NM_001042475.2:c.193G>A, p.(Asp65Asn)	P	^52^
A1422001	M	LIS	RELN	Het	Paternal	NM_005045.3:c.5351+1G>A	P	-
A1422002	M	PCH	KDM4A, KDM4A-AS1, ST3GAL3	Hom	Maternal, Paternal	[GRCh37]1p34.1(44169691_44174081)x0	VUS	-
A1422015	F	PCH	ACO2	Hom	Maternal, Paternal	NM_001098.3:c.1253C>T, p.(Pro418Leu)	VUS	-

Human reference genome used is GRCh38 unless otherwise indicated.

Cl, pathogenicity classification; F, female; Het, heterozygote; Hom, homozygote; LIS, lissencephaly; LP, likely pathogenic; M, male; P, pathogenic; PCH, pontocerebellar hypoplasia; PMG, polymicrogyria; Ref, reference; Seg, segregation; VUS, variant of uncertain significance; Zyg, zygosity.

## Discussion

Whilst individually rare, collectively, brain malformations are significant causes of epilepsy, developmental delay, intellectual disability and cerebral palsy and are a cause for termination of pregnancy. Making a genetic diagnosis has substantial implications for providing accurate genetic and prognostic counselling and limiting unnecessary investigations such as repeated brain imaging or biochemical studies. Previous genomic studies of patients with brain malformations have found a broad range of diagnostic yield from 20% for polymicrogyria^53^ to 81% for lissencephaly,^54^ although both these studies used targeted gene panels. A 2007 study using chromosome microarray and single gene sequencing showed a diagnostic rate of 19% in 113 patients with a spectrum of cortical malformations.^55^ A 2018 study using chromosome microarray and singleton ES found a diagnostic rate of 26% in 54 patients with a range of cortical malformations.^3^ CNVs detected by microarray accounted for ∼20% of the diagnoses in these cohorts, whilst these cases were screened out of the cohort in our study as a negative microarray was an inclusion criterion. Our study aimed to determine the yield of ES in a large cohort of patients with a spectrum of well-defined brain malformations, reflective of the ‘real world’ clinical practice of child neurologists and clinical geneticists.

Ten phenotypes were ascertained. The most common was polymicrogyria, accounting for 36% of the cohort, with cobblestone malformation, Joubert syndrome and subcortical band heterotopia being the least common, collectively accounting for only 6% of the cohort. We refined our initial phenotypic groups as the ascertainment progressed. Five patients had combined polymicrogyria and grey matter heterotopia, and these were separated as a phenotype. Ten patients had imaging features suggestive of a tubulinopathy, including dysgyria, dysmorphic basal ganglia, midline cerebellar dysgenesis or brainstem asymmetry.

**Figure 1:**
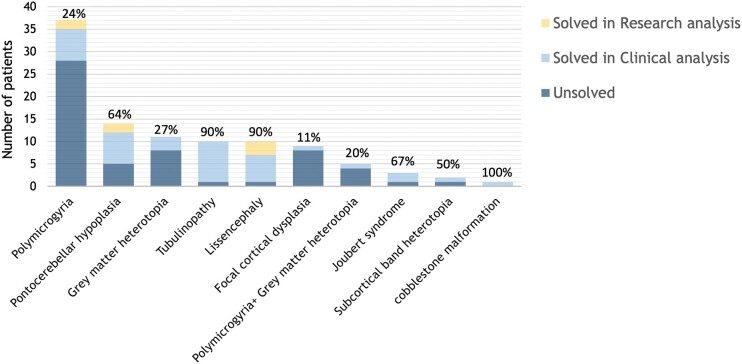
**Overall phenotype-based diagnostic yield and the relative contribution of clinical versus research analysis of exome sequence data.** Patients were grouped according to phenotype classification and the overall diagnostic yield achieved through analysis of exome data, as described in the materials and methods, and indicated by the percentage at the top of each bar. The numbers in each phenotypic group were as follows: polymicrogyria (37), pontocerebellar hypoplasia (14), grey matter heterotopia (11), tubulinopathy (10), lissencephaly (10), focal cortical dysplasia (9), polymicrogyria + grey matter heterotopia (5), Joubert syndrome (3), subcortical band heterotopia (2) and cobblestone malformation (1). Clinical analysis was restricted to phenotype-specific gene lists and extended to the Mendeliome if negative. Research analysis was performed using a tiered approach that extended to exome-wide variant analysis for unsolved patients and also included analysis of the exome data for CNVs. Patient categories are indicated below the chart and total patient numbers are shown on the *x*-axis. The number of diagnoses achieved by clinical analysis, research analysis and unsolved patients are indicated in each bar by different colours.

The diagnostic yield using clinical singleton ES was 36% (37/102). Through research follow-up incorporating data reanalysis, trio analysis and CNV analysis, the yield of likely pathogenic or pathogenic variants was improved to 43%. Diagnostic rates achieved via clinical singleton ES were highest for cobblestone malformation (1/1 = 100%), tubulinopathy (9/10 = 90%), Joubert syndrome (2/3 = 67%), lissencephaly (6/10 = 60%) and pontocerebellar hypoplasia (7/14 = 50%), acknowledging that the numbers in each group were relatively small. Diagnostic rates were lowest for polymicrogyria (7/37 = 19%), grey matter heterotopia (1/5 = 20%) and focal cortical dysplasia (1/9 = 11%). Following research reanalysis of the singleton ES data, the diagnostic rates for lissencephaly, pontocerebellar hypoplasia and polymicrogyria improved to 90% (9/10), 64% (9/14) and 24% (9/37), respectively (Fig. 1). These diagnostic rates generally reflect the existing literature from phenotype-specific studies. The one exception is cortical dysplasia, in which large cohort studies analysing brain tissue have shown diagnostic rates between 32% and 55%,^56,57^ compared with the 11% achieved in this study. This reflects the difference in yield from lymphocyte-derived DNA to that derived from resected brain samples, which enables the detection of both germline and somatic variants restricted to the dysplastic tissue. Germline causes of cortical dysplasia account for only 6–8% of cases, similar to our findings.

We limited our study to specific types of brain malformations with well-defined phenotypes and a robust list of causative genes. The strength of this approach is that it reflects clinical practice, in which accurate phenotyping precedes genomic sequencing and the selection of the gene list to interrogate in the initial genomic analysis. Our diagnostic yield is, therefore, greater than expected if we had included patients with poorly defined imaging phenotypes or malformations for which the genetic causes are unknown.

Our study highlighted the utility of periodic reanalysis of uninformative clinical exome data in genetically unsolved affected patients. Fifty-seven per cent (4/7) of the subsequently solved cases had causative variants in genes recently discovered to be associated with brain malformations; i.e. associations reported after the original diagnostic gene lists were curated. This highlights the necessity of implementing a periodic systematic reanalysis of genomic data for patients with negative results, especially prior to further pregnancies.^58^

CNV analysis using ES data accounted for 28% (2/7) of the genetic diagnoses made in the research follow-up stage. Algorithms are now available that can detect CNVs from ES data. This is valuable as the CNVs detected using these algorithms are usually relatively small multi-exon deletions that fall below the resolution of standard microarray testing used in the clinical setting. In this study, the microarray resolution was ∼0.2 Mb. Therefore, CNV analysis of the ES data is an efficient technique to identify CNVs missed by array-based methods and should be performed as a routine component of clinical ES if possible.^59,60^

The diagnostic rate increased by one patient with parent–child trio ES analysis. In the clinical stage of the study, segregation analysis was performed only on the advice of the MDT. In the research phase, the variant filtering and prioritization were less stringent compared with the clinical protocols and utilized a more extensive gene list; therefore, the variant search resulted in a high number of candidate variants that could not be accurately classified without segregation data. Furthermore, variants that may be missed in a singleton data search stand out in a trio analysis where the inheritance pattern is incorporated into the analysis. We used trio analysis to identify one additional variant and facilitated segregation analysis of other variants identified in the research follow-up stage.^61^

Studies of cortical malformations have shown that the three most common are polymicrogyria, grey matter heterotopia and cortical dysplasia.^62^ Our study demonstrates that these three malformations have the lowest diagnostic rates using ES of lymphocyte-derived DNA. This presents a problem in clinical practice as there is a need for improved diagnostic rates to provide focussed genetic counselling and family planning related to these malformations. Brain-specific somatic mosaicism is a significant cause of focal cortical dysplasia and may also play a role in other brain malformations with low diagnostic yields, including for some patients with polymicrogyria.^53^ Ultradeep sequencing from tissue sources other than lymphocyte-derived DNA may help to increase diagnostic yield for these disorders. Another reason for the low diagnostic rate for some brain malformations may be due to non-genetic causes, such as *in utero* infection. For example, *in utero* CMV infection is a well-recognized acquired cause of polymicrogyria. Although CMV infection was excluded by PCR analysis of the neonatal Guthrie card as a condition of study entry for patients with polymicrogyria, no positive CMV cases were identified. However, the CMV detection rate for this test ranges from 46% to 100%, depending on the method used and the viral load.^63^

The most likely reason for the low diagnostic rate for some malformations is that there are limited known causative gene lists and additional genetic causes remain to be discovered. After the completion of the clinical diagnostic component of this study, several new genes associated with brain malformations were reported, including four genes associated with lissencephaly^64-67^ and six with polymicrogyria.^68-73^ The increase in the diagnostic rate of polymicrogyria in the research follow-up was the result of finding causal variants in some of these newly described genes. The diagnostic yield is also influenced by the genomic assay performed. Variants in untranslated regions, promoters, introns and the mitochondrial genome and translocations, chromosomal rearrangements and repeat expansions are not typically identified and assessed using ES. To identify such variants, genome sequencing would be the test of choice.

This study highlights the importance of close collaboration of a MDT in the genetic investigation of patients with rare congenital brain abnormalities. Classification of the phenotype is the first step and requires expertise in ‘brain dysmorphology’ and the ability to accurately interpret brain MRI images. Such skills are gained through experience in imaging pattern recognition. Classification of the phenotype is then followed by determining whether a genetic or non-genetic cause is likely. If a genetic cause is expected, the appropriate genetic test must be chosen, usually beginning with chromosome microarray followed by genomic testing. Whilst trio ES only led to one additional diagnosis in our study, beginning with trio ES is preferred if possible due to time and resource saving in exome analysis and variant curation in addition to the higher diagnostic utility compared with singleton ES.^74^

Prioritization and curation of the variants found through genomic testing also benefits from the collaboration of clinicians and bioinformaticians before the final genomic report is returned to the referring clinician for discussion with the patient and their parents. The regular systematic reanalysis of the data, CNV analysis and trio ES analysis for patients with a negative result in singleton ES will improve the diagnostic rate. Genome sequencing may also improve the diagnostic rate compared with ES by detecting non-coding and CNVs, but it is still not standard of care in most clinical diagnostic services. This is likely to change as the cost of genome sequencing continues to come down. Most brain malformations have significant sequelae, so the MDT must work together to make an accurate diagnosis. The need for this interdisciplinary approach is only set to increase in importance as foetal genomics begins to influence decisions about pregnancy management.^75^

## Supplementary Material

fcae056_Supplementary_Data

## Data Availability

All supporting data in this study are available from the corresponding author on request, subject to appropriate privacy and ethical restrictions.
