# Supplementary material for: Diagnostic utility of exome sequencing followed by research reanalysis in human brain malformations
Source: Brain Commun. 2024 Feb 28;6(2):fcae056. doi: 10.1093/braincomms/fcae056 (PMC10914449; doi:10.1093/braincomms/fcae056)
Supplement: fcae056_Supplementary_Data [file fcae056_supplementary_data.docx]

**Supplementary table 1. Clinical gene lists.**

| Holoprosencephaly and septo-optic dysplasia v38.1.2 | | | | | | | | |
| --- | --- | --- | --- | --- | --- | --- | --- | --- |
| CDON | | FGFR1 | | HESX1 | | SHH | | TGIF1 |
| DISP1 | | GAS1 | | NODAL | | SIX3 | | ZIC2 |
| DLL1 | | GLI2 | | PPP1R12A | | STIL | |  |
| FGF8 | | GLI3 | | PTCH1 | | TDGF1 | |  |
| Joubert syndrome and other cerebellar malformations v38.1.0 | | | | | | | | |
| AHI1 | | CPLANE1 | | NPHP1 | | TCTN3 | | WDR81 |
| ARL13B | | CSPP1 | | NPHP3 | | TMEM138 | | WNT1 |
| ARMC8 | | FOXC1 | | OFD1 | | TMEM216 | | ZIC1 |
| B9D1 | | INPP5E | | PDE6D | | TMEM231 | | ZIC4 |
| B9D2 | | KIF7 | | RPGRIP1L | | TMEM237 | |  |
| CC2D2A | | LAMC1 | | SUFU | | TMEM67 | |  |
| CEP290 | | MKS1 | | TCTN1 | | TTC21B | |  |
| CEP41 | | NID1 | | TCTN2 | | VLDLR | |  |
| Cerebellar and Pontocerebellar hypoplasia v38.1.1 | | | | | | | | |
| AMPD2 | | EXOSC5 | | KIAA1109 | | SLC25A46 | | TUBA1A |
| AUTS2 | | EXOSC8 | | OPHN1 | | SPTBN2 | | TUBA8 |
| BCL11A | | EXOSC9 | | PCLO | | TBC1D23 | | TUBB2B |
| BRF1 | | FOXP1 | | PPP1CB | | TBCK | | TUBB3 |
| CASK | | FZD3 | | PUS3 | | TOE1 | | UFM1 |
| CEP55 | | GPAA1 | | RAB11B | | TRAPPC6B | | VLDLR |
| CHMP1A | | INTS8 | | RARS2 | | TSEN15 | | VPS53 |
| CLP1 | | ITPR1 | | RELN | | TSEN2 | | VRK1 |
| DDX3X | | KAT5 | | SEPSECS | | TSEN34 | | WDR37 |
| EXOSC3 | | KCNC3 | | SETD2 | | TSEN54 | | WDR91 |
| Lissencephaly and band heterotopia v38.1.1 | | | | | | | | |
| ACTB | | CRADD | | ISPD | | NDE1 | | TUBA1A |
| ACTG1 | | CTNNA2 | | KIF2A | | PAFAH1B1 | | VLDLR |
| ARX | | DCX | | KIF5C | | RELN | |  |
| B3GALNT2 | | DYNC1H1 | | LAMA2 | | RXYLT1 | |  |
| B3GNT2 | | EML1 | | LAMB1 | | SNAP29 | |  |
| B4GAT1 | | GMPPB | | LARGE1 | | SRD5A3 | |  |
| Cobblestone malformations v38.1.0 | | | | | | | | |
| COL3A1 | | FKTN | | POMGNT1 | | POMT1 | | RXYLT1 |
| FKRP | | LARGE1 | | POMGNT2 | | POMT2 | | TMTC3 |
| Polymicrogyria and schizencephaly v38.11 | | | | | | | | |
| ADGRG1 | DYNC1H1 | | KIF5C | | PI4KA | | SRPX2 | |
| AHI1 | EML1 | | L1CAM | | PIK3CA | | TMEM216 | |
| AKT3 | EMX2 | | LAMC3 | | PIK3R2 | | TUBA1A | |
| ARX | EOMES | | MAP1B | | RAB18 | | TUBA8 | |
| ASTN1 | FIG4 | | NDE1 | | RAB3GAP1 | | TUBB | |
| ATP1A2 | GRIN1 | | NHEJ1 | | RAB3GAP2 | | TUBB2B | |
| BICD2 | GRIN2B | | NSDHL | | RTTN | | TUBB3 | |
| COL18A1 | INTS8 | | OCLN | | SHH | | WDR62 | |
| COL4A1 | KIF1BP | | PAX6 | | SIX3 | |  | |
| Periventricular grey matter heterotopia v38.1.0 | | | | | | | | |
| ARFGEF2 | ERMARD | | FLNA | | MAP1B | | NEDD4L | |
| DCHS1 | FAT4 | |  | |  | |  | |
| Tuberous sclerosis, cortical dysplasia and hemimegalencephaly v38.1.0 | | | | | | | | |
| AKT3 | DEPDC5 | | NPRL2 | | PTEN | | TSC1 | |
| BRAF | MTOR | | NPRL3 | | STRADA | | TSC2 | |
| Microcephaly v38.1.2 | | | | | | | | |
| AKT3 | CHD4 | | MRE11 | | PQBP1 | | TPRKB | |
| ANKLE2 | CLTC | | MSMO1 | | PTPN23 | | TRAIP | |
| AP4B1 | CTCF | | NACC1 | | PUF60 | | TRAPPC12 | |
| AP4E1 | CTNNA2 | | NBN | | RAB18 | | TRAPPC6B | |
| AP4M1 | CTU2 | | NCAPD2 | | RAB3GAP1 | | TRAPPC9 | |
| AP4S1 | DNA2 | | NCAPD3 | | RAB3GAP2 | | TRIO | |
| ARCN1 | DNM1L | | NCAPH | | RAC1 | | TRIP13 | |
| ARFGEF2 | DONSON | | NDE1 | | RARS2 | | TRIT1 | |
| ASPM | DYRK1A | | NHEJ1 | | RBBP8 | | TRMT10A | |
| ATP1A2 | EFTUD2 | | NIN | | RTTN | | TSEN2 | |
| ATR | EIF2S3 | | NSD2 | | RUSC2 | | TSEN34 | |
| ATRX | FDXR | | NUP107 | | SASS6 | | TSEN54 | |
| BPTF | FOXG1 | | NUP188 | | SELENOI | | TUBB2B | |
| BRD4 | HIST1H4C | | ORC1 | | SLC1A4 | | TUBGCP6 | |
| BUB1B | IER3IP1 | | ORC4 | | SLC25A19 | | UBE3A | |
| CASK | KIF11 | | ORC6 | | SLC2A1 | | UNC80 | |
| CDK13 | KIF14 | | OSGEP | | SLC9A6 | | USP18 | |
| CDK5RAP2 | KLHL7 | | PAFAH1B1 | | STAG1 | | VRK1 | |
| CDK6 | KNL1 | | PCGF2 | | STAMBP | | WDR37 | |
| CDKL5 | LAGE3 | | PCNT | | STIL | | WDR4 | |
| CDT1 | LARP7 | | PCYT2 | | TAF2 | | WDR62 | |
| CENPE | LIG4 | | PHC1 | | TBC1D20 | | ZEB2 | |
| CENPJ | LINGO1 | | PIGH | | TBCD | | ZNF335 | |
| CEP135 | MCPH1 | | PNKP | | TCF4 | |  | |
| CEP152 | MECP2 | | POGZ | | TMX2 | |  | |
| CEP63 | MED17 | | POMT1 | | TOP3A | |  | |
| CHAMP1 | MFSD2A | | PPP1R15B | | TP53RK | |  | |

**Supplementary table 2. Tier 1 gene list.**

| A2ML1 | BRWD3 | CTSA | FIBP | IBA57 | MCPH1 | PAK3 | PAK3 | PTCH1 | SLC25A19 | TNFRSF11A | ZMYM3 |
| --- | --- | --- | --- | --- | --- | --- | --- | --- | --- | --- | --- |
| ABCC9 | BUB1B | CTSK | FIG4 | IDS | MECP2 | PANK2 | PANK2 | PTCH2 | SLC25A22 | TNFSF11 | ZNF335 |
| ACSL4 | C19orf12 | CTU2 | FKRP | IDUA | MED12 | PAX6 | PAX6 | PTCHD1 | SLC25A46 | TOE1 | ZNF41 |
| ACTB | CA2 | CUL4B | FKTN | IER3IP1 | MED17 | PCDH19 | PCDH19 | PTEN | SLC2A1 | TOP3A | ZNF423 |
| ACTG1 | CACNA1H | CYP27A1 | FLNA | IGBP1 | MEF2C | PCGF2 | PCGF2 | PTH1R | SLC9A6 | TP53RK | ZNF674 |
| ADA2 | CACNB4 | D2HGDH | FLNB | IKBKG | METTL5 | PCLO | PCLO | PTPN11 | SMARCA2 | TPP1 | ZNF711 |
| ADAR | CASK | DAG1 | FLVCR2 | IL1RAPL1 | MFSD2A | PCNT | PCNT | PTPN23 | SMARCA4 | TPRKB | ZNF81 |
| ADGRG1 | CASR | DARS1 | FMR1 | INPP5E | MID1 | PCYT2 | PCYT2 | PUF60 | SMARCB1 | TRAF7 |  |
| ADGRG1 | CBL | DCAF17 | FOXC1 | INTS8 | MIR17HG | PDE6D | PDE6D | PUS3 | SMARCE1 | TRAIP |  |
| AFF2 | CC2D2A | DCHS1 | FOXG1 | IQSEC2 | MKS1 | PDGFRB | PDGFRB | PYCR2 | SMC1A | TRAPPC12 |  |
| AGA | CCND2 | DCX | FOXH1 | ISPD | MLC1 | PDHA1 | PDHA1 | QARS | SMC3 | TRAPPC6B |  |
| AGMO | CDC6 | DDX11 | FOXP1 | ITM2B | MLYCD | PDHB | PDHB | QARS1 | SMO | TRAPPC9 |  |
| AGTR2 | CDK13 | DDX3X | FTL | ITPR1 | MPDZ | PDHX | PDHX | RAB11B | SMPD4 | TREX1 |  |
| AHI1 | CDK5 | DEPDC5 | FTSJ1 | JAM3 | MRE11 | PDP1 | PDP1 | RAB18 | SMS | TRIO |  |
| AKT1 | CDK5RAP2 | DHCR24 | FUCA1 | KAT5 | MSMO1 | PEX1 | PEX1 | RAB34 | SNAP29 | TRIP13 |  |
| AKT3 | CDK6 | DHCR7 | FZD3 | KAT6A | MTOR | PEX10 | PEX10 | RAB39B | SNX10 | TRIT1 |  |
| AKT3-IT1 | CDKL5 | DIAPH1 | GABRA1 | KAT6B | MYBPC1 | PEX12 | PEX12 | RAB3GAP1 | SON | TRMT10A |  |
| ALDH7A1 | CDKN1C | DIS3L2 | GABRD | KATNB1 | MYCN | PEX13 | PEX13 | RAB3GAP2 | SOS1 | TSC1 |  |
| AMER1 | CDON | DISP1 | GABRG2 | KCNC3 | NAA10 | PEX14 | PEX14 | RAB40AL | SOS2 | TSC2 |  |
| AMPD2 | CDT1 | DKC1 | GALC | KCNH5 | NACC1 | PEX16 | PEX16 | RAC1 | SOST | TSEN15 |  |
| AMT | CENPE | DLAT | GALNS | KCNQ2 | NAGA | PEX19 | PEX19 | RAD21 | SOX11 | TSEN2 |  |
| ANKH | CENPF | DLD | GAMT | KCNQ3 | NAGLU | PEX2 | PEX2 | RAF1 | SOX3 | TSEN34 |  |
| ANKLE2 | CENPJ | DLG3 | GAS1 | KCNT1 | NARR | PEX26 | PEX26 | RAI1 | SOX9 | TSEN54 |  |
| AP1S2 | CEP135 | DLL1 | GCDH | KCTD7 | NBN | PEX3 | PEX3 | RARS2 | SPATA5 | TSPAN7 |  |
| AP4B1 | CEP152 | DMRTA2 | GCSH | KDM5C | NCAPD2 | PEX5 | PEX5 | RASA2 | SPRED1 | TTC21B |  |
| AP4E1 | CEP290 | DNA2 | GDI1 | KDM6A | NCAPD3 | PEX7 | PEX7 | RASGRP2 | SPTAN1 | TUBA1A |  |
| AP4M1 | CEP41 | DNM1L | GFAP | KIAA1109 | NCAPH | PGK1 | PGK1 | RBBP8 | SPTBN2 | TUBA3E |  |
| AP4S1 | CEP55 | DNMT3A | GK | KIF11 | NDE1 | PHC1 | PHC1 | RBM10 | SRD5A3 | TUBA8 |  |
| APC2 | CEP63 | DOCK6 | GLB1 | KIF14 | NEDD4L | PHF6 | PHF6 | RBPJ | SRPX2 | TUBB |  |
| APP | CEP85L | DOK7 | GLDC | KIF1BP | NEU1 | PHF8 | PHF8 | RELN | STAG1 | TUBB2A |  |
| ARCN1 | CHAMP1 | DONSON | GLI2 | KIF2A | NEXMIF | PHGDH | PHGDH | RHEB | STAMBP | TUBB2B |  |
| ARFGEF2 | CHD2 | DYNC1H1 | GLI3 | KIF5C | NF1 | PI4KA | PI4KA | RIN2 | STAU2 | TUBB3 |  |
| ARHGAP31 | CHD3 | DYRK1A | GMPPB | KIF7 | NFIB | PIBF1 | PIBF1 | RIT1 | STIL | TUBB4A |  |
| ARHGEF15 | CHD4 | EED | GNAI2 | KIFBP | NFIX | PIEZO2 | PIEZO2 | RNASEH2A | STRADA | TUBG1 |  |
| ARHGEF6 | CHD7 | EEF1A2 | GNAQ | KLF8 | NHEJ1 | PIGH | PIGH | RNASEH2B | STUB1 | TUBGCP4 |  |
| ARID1A | CHD8 | EFHC1 | GNPTAB | KLHL7 | NHLRC1 | PIK3CA | PIK3CA | RNASEH2C | STXBP1 | TUBGCP6 |  |
| ARID1B | CHMP1A | EFTUD2 | GNPTG | KMT2E | NID1 | PIK3R2 | PIK3R2 | RNF125 | SUFU | TYROBP |  |
| ARL13B | CHRNA2 | EHMT1 | GNS | KNL1 | NIN | PLA2G6 | PLA2G6 | RNF135 | SUZ12 | UBE2A |  |
| ARMC8 | CHRNA4 | EIF2B3 | GOSR2 | KPTN | NIPBL | PLCB1 | PLCB1 | RNU4ATAC | SYN1 | UBE3A |  |
| ARSB | CHRNB2 | EIF2B5 | GPAA1 | KRAS | NLGN3 | PLEKHG2 | PLEKHG2 | RPGRIP1L | SYNGAP1 | UFM1 |  |
| ARX | CIT | EIF2S3 | GPC3 | L1CAM | NLGN4X | PLEKHG6 | PLEKHG6 | RPL10 | SYNJ1 | UNC80 |  |
| ASAH1 | CKAP2L | ELP1 | GPSM2 | L2HGDH | NODAL | PLEKHM1 | PLEKHM1 | RPS6KA3 | SYP | UPF3B |  |
| ASNS | CLCN4 | EML1 | GRIA3 | LAGE3 | NOL3 | PLK4 | PLK4 | RRAS | TAF2 | USP18 |  |
| ASPA | CLCN7 | EMX2 | GRIN1 | LAMA2 | NOTCH1 | PNKP | PNKP | RTTN | TAOK1 | VDAC1 |  |
| ASPM | CLN3 | EOGT | GRIN2A | LAMB1 | NOTCH3 | PNPO | PNPO | RUSC2 | TBC1D20 | VLDLR |  |
| ASTN1 | CLN5 | EOMES | GRIN2B | LAMC1 | NPHP1 | POGZ | POGZ | RXYLT1 | TBC1D23 | VPS13A |  |
| ASXL2 | CLN6 | EPG5 | GUSB | LAMC3 | NPHP3 | POLR3A | POLR3A | SAMHD1 | TBC1D24 | VPS13B |  |
| ASXL3 | CLP1 | EPM2A | HCFC1 | LARGE1 | NPRL2 | POLR3B | POLR3B | SASS6 | TBC1D7 | VPS53 |  |
| ATP13A2 | CLTC | ERMARD | HCN1 | LARP7 | NPRL3 | POMGNT1 | POMGNT1 | SCARB2 | TBCD | VRK1 |  |
| ATP1A2 | CNTNAP2 | ETFA | HDAC8 | LEMD3 | NR2E1 | POMGNT2 | POMGNT2 | SCN1A | TBCK | WASHC5 |  |
| ATP6AP2 | COL18A1 | ETFB | HEPACAM | LGI1 | NRAS | POMK | POMK | SCN1B | TCF20 | WDR37 |  |
| ATP6V0A2 | COL3A1 | ETFDH | HERC1 | LIG4 | NRXN1 | POMT1 | POMT1 | SCN2A | TCF4 | WDR4 |  |
| ATP7A | COL4A1 | EXOSC3 | HERC2 | LINGO1 | NSD1 | POMT2 | POMT2 | SCN8A | TCIRG1 | WDR45 |  |
| ATR | COL4A2 | EXOSC5 | HESX1 | LRP2 | NSD2 | PORCN | PORCN | SELENOI | TCTN1 | WDR62 |  |
| ATRIP | COX7B | EXOSC8 | HEXA | LRP5 | NSDHL | PPP1CB | PPP1CB | SEPSECS | TCTN2 | WDR73 |  |
| ATRX | CP | EXOSC9 | HEXB | LZTR1 | NSMCE2 | PPP1R12A | PPP1R12A | SETD2 | TCTN3 | WDR81 |  |
| AUTS2 | CPLANE1 | EZH2 | HGSNAT | MACF1 | NUP107 | PPP1R15B | PPP1R15B | SF3B4 | TDGF1 | WDR91 |  |
| B3GALNT2 | CPT2 | FA2H | HIST1H4C | MAGT1 | NUP188 | PPP2R5D | PPP2R5D | SGCE | TGFB1 | WNT1 |  |
| B3GNT2 | CRADD | FAM20C | HMGB3 | MAN2B1 | NXF5 | PPT1 | PPT1 | SGSH | TGIF1 | WWOX |  |
| B4GAT1 | CREBBP | FANCB | HNRNPDL | MANBA | OCLN | PQBP1 | PQBP1 | SHH | THOC2 | XK |  |
| B9D1 | CRIPT | FAT4 | HNRNPK | MAOA | OFD1 | PQBP1 | PQBP1 | SHOC2 | THOC6 | XRCC4 |  |
| B9D2 | CRPPA | FDXR | HPRT1 | MAP1B | OPHN1 | PRICKLE1 | PRICKLE1 | SHROOM4 | TMEM138 | YWHAE |  |
| BCL11A | CSPP1 | FERMT3 | HRAS | MAP2K1 | ORC1 | PRICKLE2 | PRICKLE2 | SIX3 | TMEM216 | ZDHHC15 |  |
| BICD2 | CSTB | FGD1 | HSD17B10 | MAP2K2 | ORC4 | PRKACA | PRKACA | SLC12A6 | TMEM231 | ZDHHC9 |  |
| BPTF | CTCF | FGF8 | HSD17B4 | MAST1 | ORC6 | PRKDC | PRKDC | SLC16A2 | TMEM237 | ZEB2 |  |
| BRAF | CTNNA2 | FGFR1 | HTRA1 | MBD5 | OSGEP | PRRT2 | PRRT2 | SLC17A5 | TMEM67 | ZIC1 |  |
| BRD4 | CTNNA2 | FGFR3 | HUWE1 | MBTPS2 | OSTM1 | PRUNE1 | PRUNE1 | SLC1A4 | TMTC3 | ZIC2 |  |
| BRF1 | CTNNB1 | FH | HYAL1 | MCOLN1 | PAFAH1B1 | PSAP | PSAP | SLC25A1 | TMX2 | ZIC4 |  |

**Supplementary table 3. Tier 2 gene list.**

| AC068580.4 | CCDC22 | DNAL4 | GAN | KIFC1 | MTR | PIK3C3 | RAD50 | SESN3 | TUBA4A |
| --- | --- | --- | --- | --- | --- | --- | --- | --- | --- |
| AC092143.1 | CCDC88C | DNM1 | GJB1 | KLC2 | MTRR | PIK3CB | RAD51 | SFTPC | TUBA4B |
| ACADM | CDC45 | DPP6 | GLI1 | KMT2D | MYC | PIK3CD | RALGAPB | SH2B3 | TUBAL3 |
| ACD | CDK4 | DYNC1I2 | GLIS3 | KNL1 | NCAPG | PIK3CG | RALGDS | SKI | TUBB1 |
| ACSL3 | CELSR1 | DYNC1LI1 | GLT8D1 | KSR1 | NCR2 | PIK3IP1 | RAP1A | SLC17A6 | TUBB4B |
| ACVR1B | CEP104 | DYNC1LI2 | GLT8D2 | LAMA1 | NDEL1 | PIK3R1 | RAPGEF3 | SLC22A11 | TUBB6 |
| ACY3 | CEP120 | DYNLRB1 | GMNN | LAMTOR1 | NDUFB10 | PIK3R3 | RAPGEF4 | SLC22A8 | TUBB8 |
| ADGRF3 | CEP170 | DYNLRB2 | GMPPA | LAMTOR2 | NF2 | PIK3R4 | RASD2 | SLC30A7 | TUBD1 |
| ADGRG1 | CES1 | DYNLT1 | GON7 | LAMTOR3 | NGLY1 | PIK3R5 | RASSF7 | SLC35A2 | TUBE1 |
| AKT1S1 | CETN3 | DYNLT3 | GPM6B | LAMTOR4 | NHP2 | PIK3R6 | RCAN1 | SLC9A3R2 | TUBG2 |
| AL133500.1 | CFL2 | EBF3 | GRB2 | LAMTOR5 | NID2 | PIKFYVE | RCOR1 | SMC2 | TUBGCP2 |
| AMER3 | CHN1 | EFNB3 | GRIN3A | LARGE1 | NLE1 | PKD1 | RGL1 | SMC4 | TUBGCP3 |
| ANO5 | CIC | EGFR | GXYLT1 | LARGE2 | NOL9 | PKD2 | RICTOR | SNRPE | TUBGCP5 |
| ANP32A | CLIP1 | EIF2AK4 | GXYLT2 | LARP1B | NPHP4 | PKHD1 | RMI2 | SNX14 | TXNDC15 |
| AP001273.2 | CLUAP1 | EIF4B | HHAT | LOX | NTN1 | PLAA | RNF113A | SPOP | UBA5 |
| ARAF | CMIP | EIF4E | HHIP | LPIN1 | NUDCD2 | PLXDC1 | RNF149 | SPRY1 | UNC119 |
| ARF1 | CNDP2 | EIF4EBP1 | HOXB1 | LRP8 | NUDCD3 | PMPCA | ROBO1 | STK25 | UNC119B |
| ARFGEF1 | CNKSR1 | EMG1 | HSPG2 | LRPAP1 | NUP37 | PNPLA2 | ROBO3 | STK36 | VANGL1 |
| ARHGEF7 | CNKSR2 | EML4 | HSPH1 | LRRCC1 | NUP43 | POC1A | RPS6KA1 | STRADB | VANGL2 |
| ARHGEF9 | COPB2 | ERLEC1 | HYLS1 | MAFB | ODC1 | POFUT1 | RPS6KB1 | SYVN1 | VCP |
| ARL3 | CPLANE1 | ESR1 | IFT172 | MAG | OLIG2 | PPP2CA | RPTOR | TBR1 | VWA3B |
| ARMC9 | CTSD | EVC2 | IFT88 | MAP11 | ORC2 | PPP2R1A | RRAGA | TBXT | WDFY3 |
| ARNT2 | CTU1 | EXOC3L2 | IGF1 | MAP1A | ORC5 | PREX1 | RRAGB | TCL1A | WDPCP |
| ASH1L | CYBRD1 | EXOC4 | IHH | MAPK3 | OS9 | PREX2 | RRAGC | TELO2 | WDR24 |
| ATG5 | DAB1 | EXOSC1 | IL21 | MAPKAP1 | PAFAH1B2 | PRKAA1 | RRAGD | TMEM107 | WDR59 |
| ATP1A3 | DACT1 | EXOSC2 | INO80 | MAPT | PAFAH1B3 | PRKAA2 | RTEL1 | TMEM17 | WDR63 |
| B3GNT2 | DCC | EXOSC4 | INVS | MAST3 | PARD3 | PRKAB1 | RXYLT1 | TMEM246 | WIF1 |
| B4GALT1 | DCLK2 | EXOSC6 | ITGB6 | MATN4 | PCDH12 | PRKAB2 | RYK | TMEM80 | XRCC1 |
| BICD1 | DCLRE1B | EXOSC7 | ITPR2 | MCM3AP | PCDHA4 | PRKAG1 | RYR1 | TNIP2 | XXYLT1 |
| BLM | DCTN2 | EZR | ITPR3 | MCM5 | PCDHA6 | PRKAG2 | RYR2 | TNNC1 | ZBTB7A |
| BOC | DDR2 | F3 | KATNA1 | MDM2 | PCF11 | PRKAG3 | RYR3 | TOP2B | ZFYVE26 |
| BORCS5 | DDX59 | FARP2 | KCTD20 | METTL1 | PCM1 | PRKCA | SCN3A | TRIM36 | ZNF219 |
| BTBD10 | DEPDC1 | FBRS | KIAA0556 | MFNG | PCOLCE | PRR5 | SCRIB | TRIM37 |  |
| BUB1 | DEPDC1B | FBRSL1 | KIAA0586 | MGAT1 | PCSK7 | PRR5L | SCYL2 | TRRAP |  |
| C12orf57 | DEPDC4 | FBXL19 | KIAA0753 | MICALL2 | PDK1 | PSAT1 | SDF2 | TTC3 |  |
| C2CD3 | DEPDC7 | FGFR2 | KIAA1279 | MIOS | PEX6 | PSPH | SDF2L1 | TTI1 |  |
| C7orf26 | DEPTOR | FILIP1 | KIAA1549 | MKKS | PGR | PTF1A | SEC13 | TTI2 |  |
| CAB39 | DERL2 | FKBP2 | KIF1A | MKLN1 | PIGS | PTK2 | SEH1L | TUBA1B |  |
| CACNA1B | DHH | FOXP2 | KIF20A | MLST8 | PIK3C2A | PXN | SEL1L | TUBA1C |  |
| CACNA1E | DIS3 | FOXP4 | KIF21A | MTHFD1 | PIK3C2B | QRFPR | SESN1 | TUBA3C |  |
| CACNB1 | DNAH11 | FRS3 | KIF3A | MTHFR | PIK3C2G | RAB23 | SESN2 | TUBA3D |  |
